# Investigation of laser wavelength effect on the ablation of enamel and dentin using femtosecond laser pulses

**DOI:** 10.1038/s41598-023-47551-5

**Published:** 2023-11-17

**Authors:** Ludovic Rapp, Steve Madden, Julia Brand, Ksenia Maximova, Laurence J. Walsh, Heiko Spallek, Omar Zuaiter, Alaa Habeb, Timothy R. Hirst, Andrei V. Rode

**Affiliations:** 1 Laser Physics Centre, Department of Quantum Science and Technology, Research School of Physics, Australian National University, Canberra, ACT 2600 Australia; 2 The University of Queensland School of Dentistry, Herston, QLD 4006 Australia; 3 Faculty of Medicine and Health, The University of Sydney School of Dentistry, Surry Hills, NSW 2010 Australia; 4 Dentroid Pty Ltd, Canberra, ACT 2601 Australia

**Keywords:** Dentine, Enamel, Ultrafast lasers

## Abstract

We investigated the effect of femtosecond (fs) laser ablation of enamel and dentin for different pulse wavelengths: infrared (1030 nm), green (515 nm), and ultra-violet (343 nm) and for different pulse separations to determine the optimal irradiation conditions for the precise removal of dental hard tissues with the absence of structural and compositional damage. The ablation rates and efficiencies were established for all three laser wavelengths for both enamel and dentin at room temperature without using any irrigation or cooling system, and the surfaces were assessed with optical and scanning electron microscopy, optical profilometry, and Raman spectroscopy. We demonstrated that 515 nm fs irradiation provides the highest rate and efficiency for ablation, followed by infrared. Finally, we explored the temperature variations inside the dental pulp during the laser procedures for all three wavelengths and showed that the maximum increase at the optimum conditions for both infrared and green irradiations was 5.5 °C, within the acceptable limit of temperature increase during conventional dental treatments. Ultra-violet irradiation significantly increased the internal temperature of the teeth, well above the acceptable limit, and caused severe damage to tooth structures. Thus, ultra-violet is not a compatible laser wavelength for femtosecond teeth ablation.

## Introduction

The use of femtosecond lasers in dentistry is a relatively new treatment approach for a variety of dental procedures. The increasing popularity results from their ability to precisely ablate tissue without causing thermal damage. Operators can precisely remove dental hard tissues using ultra-short pulses of light, with pulse durations in the femtosecond (1 fs = 10^–15^ s) range, offering several advantages over traditional lasers and rotating dental tools. Femtosecond lasers can remove hard tissue with extremely high precision^1–3^, permitting dentists to perform tooth-cutting procedures with a higher level of accuracy than conventional treatments. Femtosecond laser ablation processes do not generate vibration reducing pain or discomfort for patients. The lack of excess heat eliminates thermal damage to the surrounding tissues. Patients are increasingly seeking out minimally invasive dental treatments with less pain and discomfort and shorter recovery times. Femtosecond lasers are a promising technology that can help improve the patient experience and care outcomes.

Currently, dentists remove hard tissue with high-speed rotary drills (the most common method) and by thermo-mechanical ablation using middle infrared erbium or holmium lasers (still in very limited use)^4^. Both methods can create micro-cracks in the tooth abetting the spread of recurrent tooth decay. Neither erbium nor holmium lasers can cut metals or ceramic fillings, and they cannot match the speed (0.5 mm^3^/s) and cutting quality of mechanical drills^4–6^.

In the last 20 years, femtosecond lasers have been more and more utilized for cataract surgery, ear surgery, and tattoo removal^7^. For dentistry, these lasers can overcome the drawbacks of traditional treatment methods^8,9^. Precisely focused ultrashort laser pulses, lasting just a few hundred femtoseconds each, are so short that almost no heat or shock is transferred into the material resulting in virtually no damage to surrounding tissue. In past work, we have demonstrated that femtosecond laser pulses can rapidly drill into the enamel and dentin of extracted healthy human permanent teeth to create precise features with minimal temperature increase inside the tooth and no changes in the chemical composition of the treated surfaces^3^. No cracking occurred in the teeth from the laser ablation process. Moreover, the laser treatment was performed without a water spray for cooling—the most common method of cooling when using traditional rotating burs for enamel removal. The femtosecond laser pulses produced very smooth surfaces, with precise depth control, as only a very thin layer of material is removed with each pulse (< 1.5 µm), while millions of pulses per second can be delivered, thus providing the necessary speed^10^.

Enamel and dentin are the two main dental tissues targeted for laser ablation in various dental procedures, such as removing decay and preparing the tooth for placing permanent restorations. The efficiency of laser ablation for enamel and dentin depends on several factors: the laser wavelength, the pulse duration, the energy density, and the repetition rate^11–15^. These parameters can be adjusted to optimize the ablation process for specific dental procedures and tissues. The laser wavelength is particularly important as it determines the absorption characteristics of the tissue and affects the level of tissue ablation and the quality of the resulting ablation. The absorption coefficient of enamel and dentin increases with decreasing laser wavelength^16^. Therefore, using shorter wavelengths (such as blue or ultraviolet) may result in more efficient ablation of dental tissues than longer wavelengths, such as infrared. However, shorter wavelengths can cause more tissue damage due to higher photon energy. In addition, dentin’s higher absorption coefficient compared to that of enamel means that dentin may be more efficiently ablated than enamel at the same laser wavelength, as we already observed for near-infrared (1030 nm)^3^. However, dentin also contains more protein and water than enamel, which can absorb laser energy and reduce ablation efficiency.

In this paper, we investigated the effect of three laser wavelengths (near infrared (IR: 1030 nm), green (green: 515 nm), and ultraviolet (UV: 343 nm)) on the ablation of healthy enamel and dentin using the femtosecond regime and determined the most efficient parameters for safe laser ablation of teeth. By understanding how wavelength and other different laser parameters affect tissue ablation, future work can optimize the use of femtosecond lasers for dental procedures, leading to better patient outcomes.

## Methods

### Teeth

Intact health human permanent teeth, collected with institutional human ethics approval, were used to study the effects of the femtosecond laser. They were selected from a collection of extracted teeth removed for orthodontic purposes and were used intact or were sectioned horizontally with a precision diamond saw (Buehler, Lake Bluff, IL, USA). Remnants of dental pulp tissue were removed. After preparation, the teeth were sterilized by gamma irradiation (25 kGray, Steritech, Narangba, Qld, Australia) and stored in a dry state. They were rehydrated with distilled water for 24 h before laser processing so that their water content was restored to the normal fully hydrated state.

### Experimental setup

The femtosecond laser processing experiments for the enamel and the dentin of the teeth were performed with a Carbide 40 W femtosecond laser (CB3-40W from Light Conversion, Lithuania). The laser wavelengths were 1030, 515, and 343 nm, and the pulse duration was set to 275 fs. The repetition rate was 100 kHz, and the maximum pulse energy was 400 µJ. The laser pulses were focused on the sample with a telecentric f-Theta femtosecond scanning lens (Vonjan Technology, Germany). A dual-axis galvoscanner (Thorlabs, USA) was used to scan the laser beam across the teeth in the single-pulse scanning regime (with well-separated laser spots on the target surface) or with pulse overlaps. The focal spot diameter at FWHM was ~ 16 µm at 1030 nm, and 10 µm at 515 and 343 nm. The steps between scanner lines were set at 5 µm. The laser fluence was varied by attenuating the laser power. In-house written LabView software controlled the laser beam scanning characteristics, such as scanning speed, inter-shot, inter-scan line distance, and two-dimensional (2D) scanning pattern design. A plume extraction system (Kemper, Germany) with an exhaust capacity of 950 m^3^/h was used to collect all vapors that were generated. All experiments were undertaken in ambient air at room temperature.

### Optical microscopy and profilometry

To determine the ablation thresholds, rates, and efficiencies, grooves were created in enamel and dentin, varying the laser fluence but keeping the same total energy deposited per groove. The ablated surfaces were observed with an optical microscope. Surface texture, roughness and depth of the ablated surface were recorded by using an optical profiler (Veeco Wyko NT9100, Bruker, USA) with a 5 × and 20 × objective and a 0.55 × field-of-view multiplier. The vertical scanning interferometry (VSI) mode was used. Surface features were assessed for structural changes by scanning electron microscopy at magnifications up to 5000 × using a Zeiss UltraPlus FESEM (Zeiss, Germany), with an accelerating voltage of 10 kV and a sample working distance of 2–5 mm. The samples were sputter coated with carbon prior to examination.

### Raman micro-spectroscopy

An InVia Reflex Raman spectrometer (Renishaw, UK) was used to investigate the possible chemical changes in tooth structure before and after laser treatment. Spectra were obtained with a 785 nm near-infrared diode laser focused on the tooth using a 20 × lens (with a 1.15 mm working distance). A grating with 1200 lines/mm was used for all measurements, and the spectra were collected on a Peltier-cooled CCD detector. Three scans with an acquisition time of 20 s were undertaken over a spectral range of 300–1800 cm^–1^ for dentin and enamel, using a laser power of 3.8 mW. Calibration used the 520.7 cm^–1^ peak of a silicon reference. The raw spectra were baseline-corrected with BSpline software interpolation, then smoothed with the Savitzky-Golay filter of polynomial order 3 and 24 points, then normalized to the 959 cm^–1^ peak, and curve-fitted with Gaussian peak profiles to precisely determine the peak positions.

### Temperature measurements

Temperature changes during laser processing were recorded by inserting a 0.5 mm diameter miniature bead thermocouple inside the dental pulp chamber of each tooth. The bead was covered with heat conductive heatsink compound before being fixed into place, to ensure that heat transfer occurred from the dentin. The position of the thermocouple was against the dentin directly opposite the point of laser processing, to measure maximal thermal changes^7^. Laser ablation was undertaken for 5 min in a square shape of 4.5 × 4.5 mm^2^, during which time data were collected using a data logger (Lascar Electronics, Hong Kong), so that the maximum temperature change could be calculated. The number of scans was 200 to achieve the 5 min period of ablation using a 100 kHz laser repetition rate. The experiments were repeated on several intact teeth. The experiments were then repeated with compressed air blowing directed onto the laser processing area at 14 m/s giving a flow rate of 14.4 L/min.

### Statement

All methods were carried out in accordance with relevant guidelines and regulations, including for biosafety and laser safety. Human permanent teeth, collected with institutional human ethics approval, were used to study the effects of the femtosecond laser. Ethical approval for the collection of extracted teeth was obtained from the Medical Research Ethics Committee at the University of Queensland, for the study of laser tooth preparation^5^. The teeth were collected with approval from adult subjects who required tooth extraction as part of their comprehensive dental treatment. The informed consent was obtained from all subjects and/or their legal guardian(s). The teeth were cleaned of debris and sterilised by gamma irradiation (25 kGy) in line with institutional biosafety approvals, so that they could be stored dry until used in the current studies.

## Results and discussion

### Ablation thresholds, rates, and efficiencies

**Figure 1:**
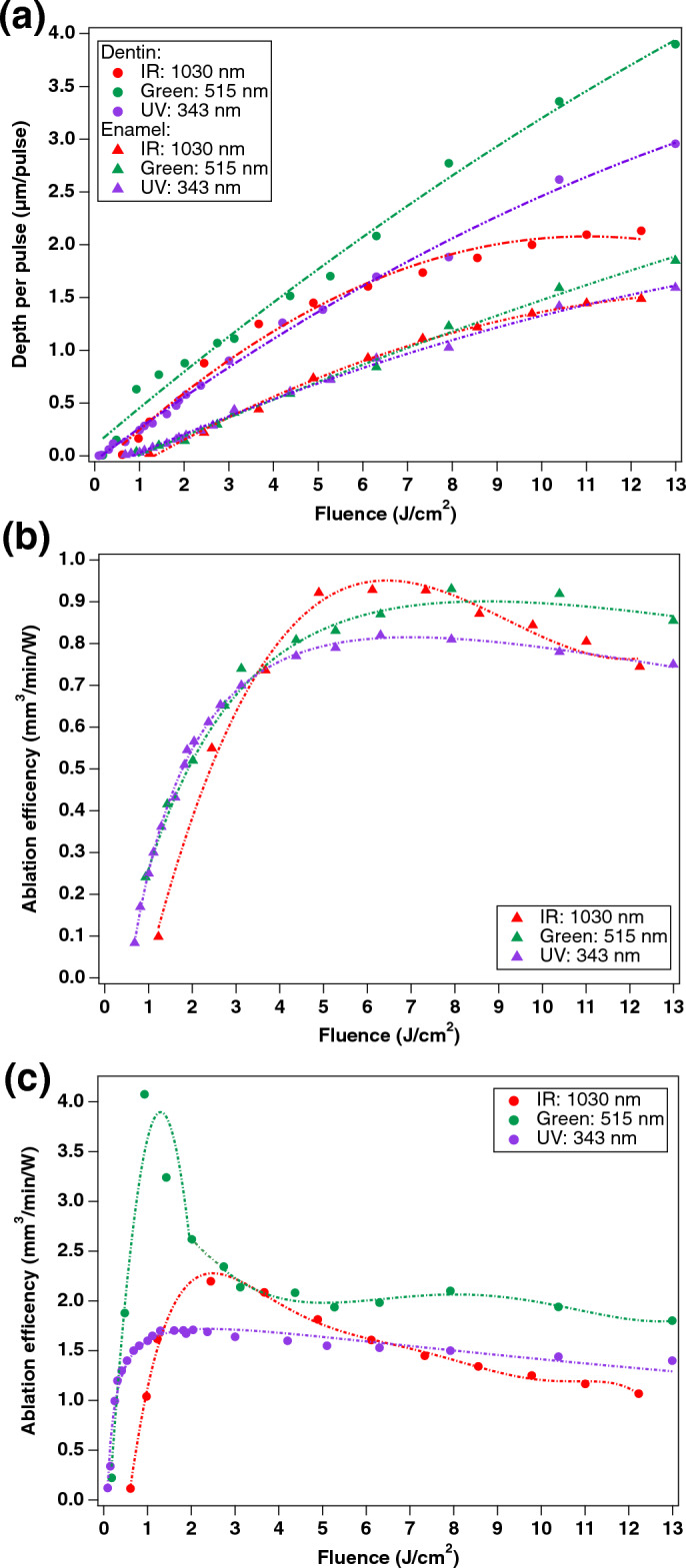
Femtosecond laser ablation of enamel and dentin for IR (1030 nm), Green (515 nm) and UV (343 nm) laser wavelengths: (**a**) Depth per pulse, Ablation efficiencies for (**b**) enamel and for (**c**) dentin.

Determining ablation thresholds, rates, and efficiency is essential for optimizing femtosecond laser material processing and ensuring consistent results. The ablation threshold is the minimum laser fluence required to start the ablation process, and it is one of the most important parameters in laser material processing. The ablation rate (i.e. the amount of material removed per unit of time) is dependent on the laser fluence and pulse duration, while the efficiency of ablation considers the ablation rate and the laser power used. We ablated a series of grooves, varying the laser fluence between the groove for each of the three laser wavelengths. We used a saw-tooth line drive waveform scanning approach in the single-shot-per-spot regime to ensure that no thermal accumulation may result from pulse overlaps. The results are presented in Table 1 and Fig. 1.

**Table 1:** Ablation threshold and optimal ablation efficiencies in enamel and dentin for femtosecond laser processing at 1030 nm, 515 nm, and 343 nm.

	IR (1030 nm)	Green (515 nm)	UV (343 nm)
Enamel			
Ablation threshold (in J/cm^2^)	1.1 ± 0.1	0.85 ± 0.05	0.7 ± 0.1
Optimal ablation efficiency (in mm^3^/min/W)	0.9 ± 0.1 at (4.5–8.5) J/cm^2^	0.9 ± 0.1 at (7.0–9.0) J/cm^2^	Damage to enamel
Dentin			
Ablation threshold (in J/cm^2^)	0.6 ± 0.1	0.20 ± 0.05	0.10 ± 0.05
Optimal ablation efficiency (in mm^3^/min/W)	1.74 ± 0.05 at (4.5–7.0) J/cm^2^	4.00 ± 0.05 at 0.9 J/cm^2^	Damage to dentin

UV irradiation had the lowest ablation threshold, at 0.7 ± 0.1 J/cm^2^ for enamel and 0.10 ± 0.05 J/cm^2^ for dentin, followed by green with 0.85 ± 0.05 J/cm^2^ (enamel) and 0.20 ± 0.05 J/cm^2^ (dentin), and then IR irradiation at 1.1 ± 0.1 J/cm^2^ (enamel) and 0.6 ± 0.1 J/cm^2^ (dentin). The optimal regime for ablation of enamel in a single pulse per shot regime was similar for both IR and green irradiation, with 0.9 ± 0.1 mm^3^/min/W in the range of (4.5–8.5) J/cm^2^ for IR and (7.0–9.0) J/cm^2^ for green.

The ablation efficiency of dentin was almost two times higher for IR with 1.74 ± 0.05 mm^3^/min/W, and almost 4.5 times higher for green irradiation with 4.00 ± 0.05 mm^3^/min/W. Green irradiation was the most effective for the ablation of both enamel and dentin, with an ablation rate ~ 2.3 times higher than IR for dentin.

UV caused visually discernible modification of the grooves for all tested fluences, thus the optimal ablation efficiencies are not presented in Table 1. Figure 1 presents the evolution of the depth per pulse and the ablation efficiencies as a function of the laser fluence. While the highest ablation rate can be achieved using green irradiation, this requires dynamic control over the fluence when crossing the enamel and dentin boundary to maintain the highest efficiency.

**Figure 2:**
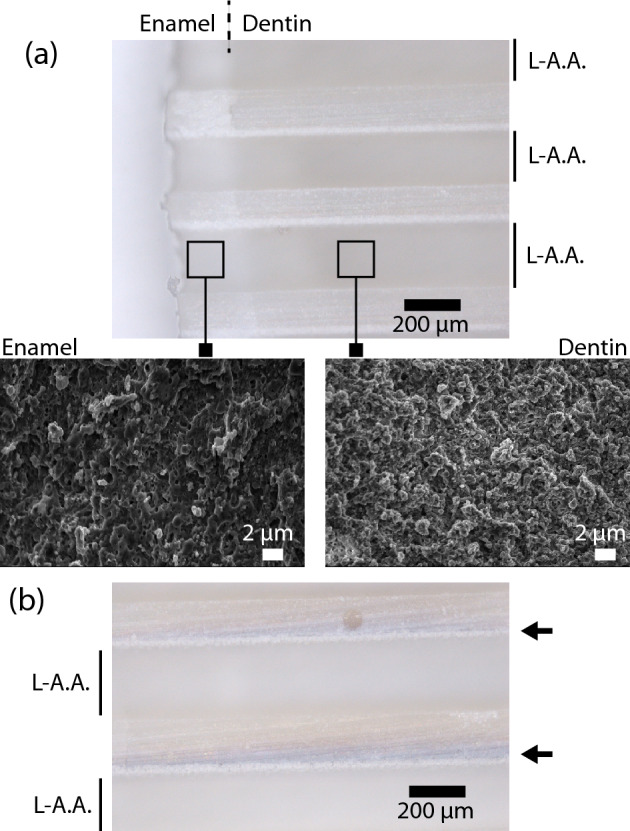
(**a**) Optical microscopy image of the femtosecond laser processed grooves in a slice of teeth with enamel and dentin in IR (1030 nm), similar observations were found for green (515 nm) irradiation). Inset images show high-power views of the enamel and dentin floor regions by scanning electron microscopy, and (**b**) charring observed on dentin grooves with 75% of pulse overlap and above, indicated by the arrows. *L-A.A*. laser-ablated area.

Analysis by optical microscopy and by scanning electron microscopy of the ablated grooves in enamel and dentine revealed no damage to the tooth structure around the grooves and on the groove floors, and very precise cuts in the enamel and dentin, as seen in Fig. 2a, for both IR and green wavelengths. The grooves were investigated by scanning electron microscopy and confirmed crack-free preparations in teeth, as also observed in previous studies^17–19^. However, grooves with UV irradiation had carbonization and charring on the walls and floors, as shown in Fig. 2b.

### Effect of pulse overlap

The overlap of laser pulses can have a marked effect on the results of ablation. The overlap refers to the interval between two consecutive laser pulses during the scanning, and it can affect efficiency, quality, and precision. If the overlap between laser pulses is too large, the material may be subject to excessive heating, which can result in damage to the material or changes in its physical properties. This occurs because the energy from one pulse is absorbed by the material and this causes a temperature rise that is not completely dissipated before the next pulse arrives, leading to cumulative heat effects. Precise control of laser pulse overlap is essential to achieve the desired results. This can be done by adjusting the repetition rate of the laser pulses or the scanning speed of the laser beam.

**Figure 3:**
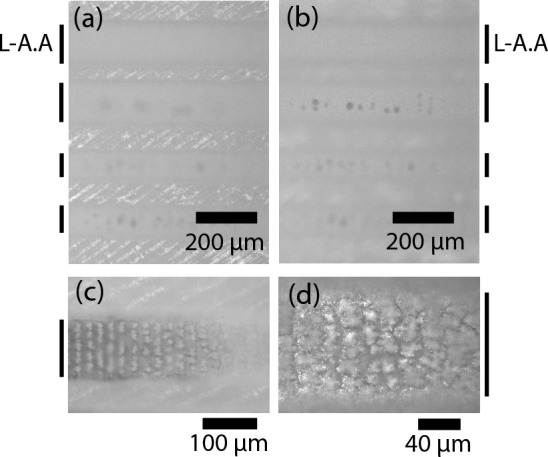
Optical microscopy image of the femtosecond laser processed grooves in the dentin of a sliced of teeth using femtosecond UV (343 nm) irradiation: (**a**) top and (**b**) floor of the laser ablated grooves presenting charring; (**c**) charring observed with 75% of pulse overlap and (**d**) magnification of the groove’s floor. *L-A.A*. laser-ablated area.

Grooves in enamel and dentin were prepared by varying the overlap between the laser pulses. Overlaps of 25, 50, 75, and 90% of the spot diameter were tested. For both IR and green, in enamel and dentin, no damage was observed by optical microscopy for up to 50% of pulse overlap. Charring started to occur with 75% overlap from 2.5 J/cm^2^, as seen in Fig. 2b. At 90% overlap, damage was observed for all fluences for both enamel and dentin. For the UV wavelength, all processed grooves showed charring of the walls and floors of the grooves, Fig. 3.

### Investigation of structural modifications by Raman spectroscopy

**Figure 4:**
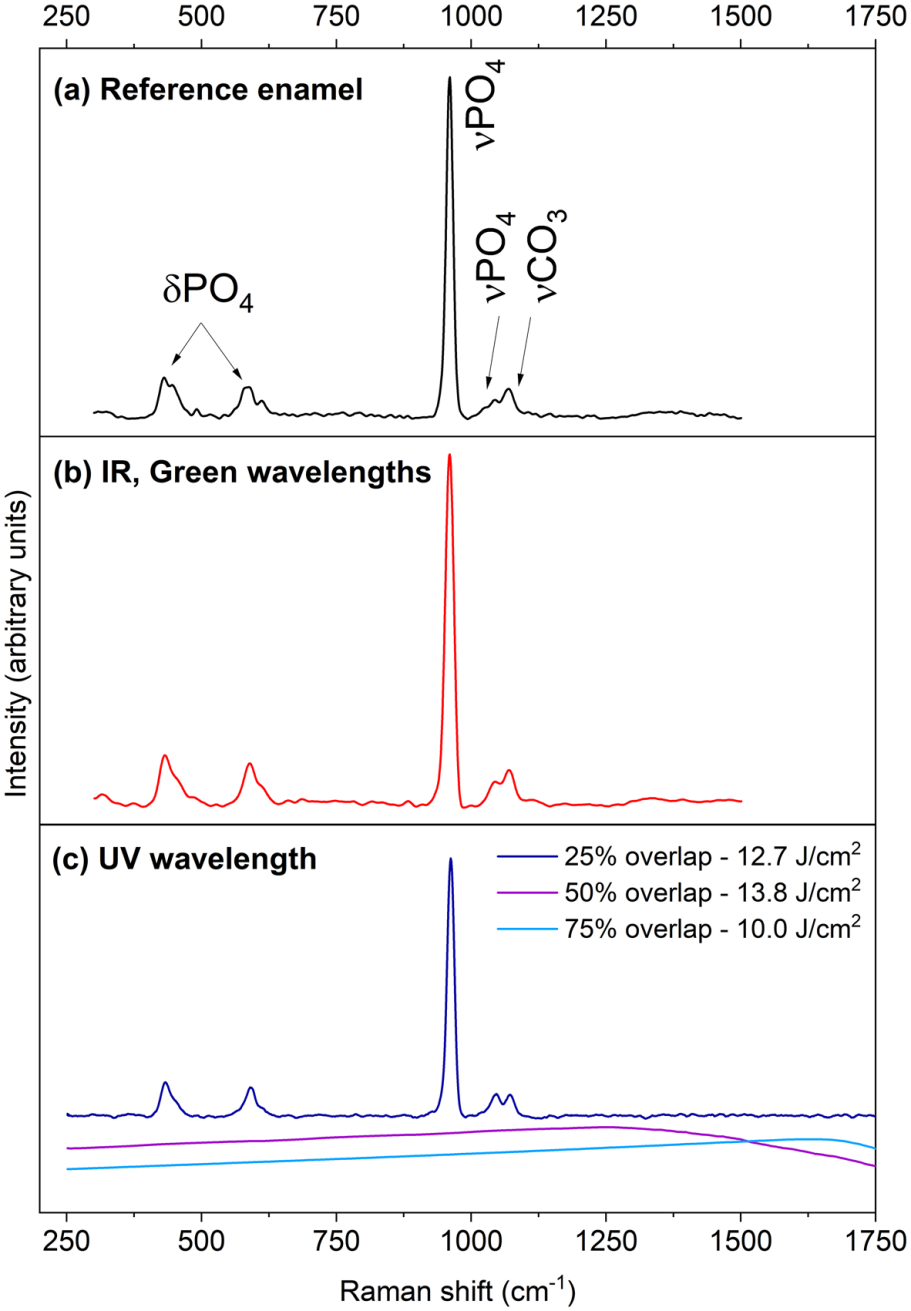
Raman spectroscopy of femtosecond laser processing of enamel at the tested wavelengths: (**a**) reference spectrum of enamel with peak attributions from^3^ (δ: bending; ν: stretching), (**b**) spectra for IR (1030 nm) and green (515 nm) irradiations at all tested fluences, showed similar behavior, and (**c**) spectra for UV (343 nm) irradiation at different fluences and overlap.

Raman spectroscopy was performed on the walls and floors of the laser-processed grooves^20–23^. Figure 4 presents the Raman spectra taken on unprocessed enamel (Fig. 4a) and enamel irradiated at different wavelengths (Fig. 4b and c). Attributions of the peaks were done elsewhere^3^. Briefly, the symmetric stretching of phosphate units (PO_4_) in apatite is visible at 959 cm^–1^. Additional vibrations of PO_4_ can be observed at 427 cm^–1^ and 584 cm^–1^ (bending) and at 1041 cm^–1^ (antisymmetric stretching). The peak at 1070 cm^–1^ was attributed to the symmetric stretching of carbonate units (CO_3_), overlapping with the stretching of PO_4_. After irradiation with the IR and green wavelengths, no structural change was observed in enamel for all tested fluences and for pulse overlaps from a single shot regime to 50% overlaps. No structural change was observed for UV irradiation in the single pulse regime and at 25% overlap up to 12.7 J/cm^2^. Above this value, all peaks disappeared from the spectra, as illustrated in Fig. 4c. At 75% pulse overlap and fluences above 10 J/cm^2^, charring was observed with a complete loss of features on the Raman spectra. This indicates severe damage to the enamel, and those fluences and high pulse overlaps should be avoided to preserve the structural integrity of the material.

**Figure 5:**
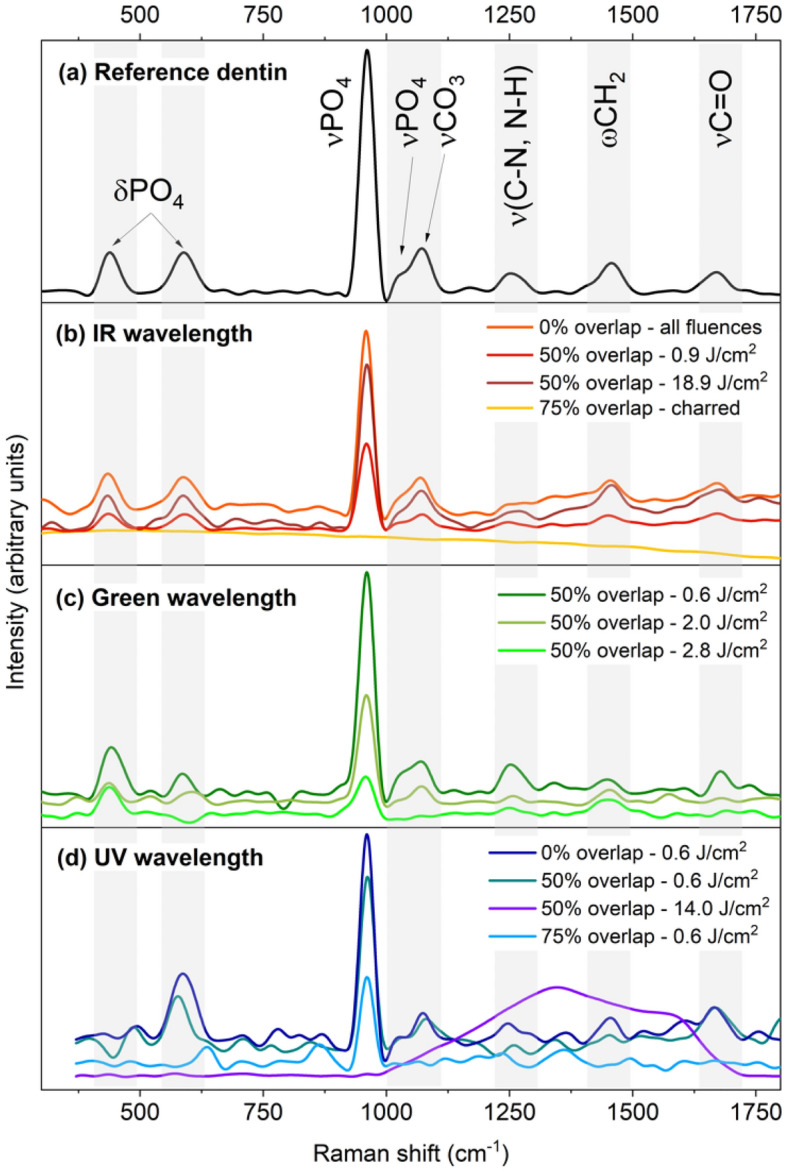
Raman spectroscopy of femtosecond laser processing of dentin at the three tested wavelengths: (**a**) reference spectrum of dentin with peak attributions from^3^ (δ: bending; ν: stretching; ω: wagging), (**b**) spectra for IR (1030 nm) irradiation at various fluences and overlap, (**c**) for green (515 nm) irradiation at different fluences and 50% overlap, and (**d**) for UV (343 nm) irradiation at different fluences and overlap. Grey bands indicate the various peak positions for clarity.

Figure 5 presents the Raman spectra taken on unprocessed dentin (Fig. 5a) and dentin irradiated at different wavelengths (Fig. 5b to d). Dentin exhibits additional organic peaks at 1240 cm^–1^, attributed to a mix of C–N stretching and N–H bending vibrations in amide III, at 1455 cm^–1^ attributed to the wagging of CH_2_, and at 1665 cm^–1^ attributed to the in-plane stretching of carbonyl in peptide bonds^3^. The other bands are artifacts from the low signal-to-noise ratio and baseline correction. For clarity, grey bands indicate where the peaks are located. After irradiation with the IR wavelength (Fig. 5b), no changes were observed on the spectra with no pulse overlap at all tested fluences, or at 50% pulse overlap. Structural modifications of the dentin were visible for all laser fluences at 75% overlap (visible charring). No characteristic peaks were observed on the spectrum at this overlap. For green irradiation, changes in peak intensity ratios were observed even at the low fluence of 0.6 J/cm^2^ for the bending of PO_4_ units (under 700 cm^–1^). Additional disappearances or broadening of peaks in the dentin spectra are observed at laser fluences above 2.0 J/cm^2^ for 50% overlap. In particular, the organic peaks in the 1200–1700 cm^–1^ range disappear, and the intensity of the main PO_4_ feature at 959 cm^–1^ decreases significantly. Hence, to avoid damage, pulse overlaps of 50% and above should be avoided. The dentin can be safely laser processed for all tested laser fluence with no pulse overlap at all (i.e. a single pulse regime) and up to 50% overlap for IR irradiation and for green irradiation below 2.0 J/cm^2^.

For UV irradiation (Fig. 5d), structural changes occurred even at the low fluence of 0.6 J/cm^2^ in the single pulse regime (with no pulse overlap). A decrease in the signal-to-noise ratio was observed, and the peaks were poorly resolved. The disappearance of organic peaks above 1200 cm^–1^ and PO_4_ bending at 427 cm^–1^ was noted. At 50% of pulse overlap, damage occurred in dentin from 0.6 J/cm^2^ as illustrated by the disappearance of the organic peaks above 1200 cm^–1^, the bending of PO_4_/carbonate peaks, and stretching (below 600 cm^–1^ and around 1000 cm^–1^). At a laser fluence of 14.0 J/cm^2^ and above, the phosphate and organic peaks completely disappeared, and broad bands appeared in the 1200–1600 cm^–1^ range indicating carbonization. For 75% of pulse overlap, severe damage was noted, resulting in complete loss of the Raman features in the spectra. Overall, with the UV wavelength, the poor resolution of the Raman spectra and loss of the Raman signatures of both enamel and dentin indicates that UV irradiation is highly damaging to healthy tooth structures, even at laser fluences below the ablation threshold of dentin, and thus UV should be avoided for the laser processing of teeth.

Overall, Raman spectroscopic studies revealed that IR and green were the most suitable wavelengths for tooth ablation. IR and green could safely process healthy tooth structure, avoiding structural damage in enamel and dentin when the laser pulse overlap was below 50%. However, a laser fluence below 2.0 J/cm^2^ must be used for green irradiation to prevent any damage to the dentin, which also corresponds to the optimal ablation efficiency for green irradiation. UV irradiation was not suitable for tooth ablation in all the tested conditions.

### Investigation of temperature changes inside teeth during laser processing

Elevations in temperature inside the pulp chamber (nerve canal) of the tooth during laser processing are presented in Table 2 for all laser wavelengths for two different fluences: 3.0 and 6.0 J/cm^2^, giving an effective average laser power of 0.5 W and 1 W, respectively. No external cooling was applied to the tooth during the experiment. A temperature increase below the maximum allowable limit of 5.5 °C^24^ was seen for both IR and green, for all tested pulse overlaps at a fluence of 3.0 J/cm^2^. At a fluence of 6.0 J/cm^2^, the temperature increase after IR irradiation was below the threshold, for 0 and 25% of pulse overlap, but overpassed the threshold by 1.5 degrees for 50% overlap and by 2.5 degrees for 75% overlap. On the contrary, the temperature increase for green irradiation at 6 J/cm^2^ was above the limit for all tested degrees of overlap. UV irradiation caused a temperature increase above the limit for all tested conditions, especially when at 6.0 J/cm^2^.

**Table 2:** Temperature change inside teeth for femtosecond laser processing at 1030, 515, and 343 nm in function of the pulse overlap during scanning and for 2 laser fluence.

Laser wavelength	Laser fluence (in J/cm^2^)	Pulse overlap (in %)	Temperature increase (in °C)
IR (1030 nm)	3.0 ± 0.1	0	4.0 ± 0.5
		25	4.0 ± 0.5
		50	4.0 ± 0.5
		75	4.5 ± 0.5
	6.0 ± 0.1	0	5.0 ± 0.5
		25	5.5 ± 0.5
		50	7.0 ± 0.5
		75	8.5 ± 0.5
Green (515 nm)	3.0 ± 0.1	0	5.5 ± 0.5
		25	5.5 ± 0.5
		50	5.5 ± 0.5
		75	5.5 ± 0.5
	6.0 ± 0.1	0	10.5 ± 0.5
		25	11.0 ± 0.5
		50	12.5 ± 0.5
		75	12.5 ± 0.5
UV (343 nm)	3.0 ± 0.1	0	7.0 ± 0.5
		25	7.5 ± 0.5
		50	8.5 ± 0.5
		75	8.5 ± 0.5
	6.0 ± 0.1	0	22.0 ± 0.5
		25	24.0 ± 0.5
		50	26.0 ± 0.5
		75	28.5 ± 0.5

The same experiments were reproduced with a flow of compressed air directed to the ablation region on the tooth. All tested laser fluences and pulse overlaps showed a temperature increase below 0.5 °C for both enamel and dentin for all three laser wavelengths.

## Conclusion

We investigated the impact of laser wavelength on the ablation of enamel and dentin using a femtosecond laser system. UV (343 nm) showed the lowest ablation threshold for both enamel and dentin. However, it caused severe damage to tooth structures resulting in charring and profound alterations in the Raman spectra. Consequently, the use of UV laser wavelengths for femtosecond laser tooth ablation is not recommended due to its detrimental effects.

For enamel ablation, both IR and green laser wavelengths have a comparable ablation efficiency when used in the single pulse regime, reaching 0.9 ± 0.1 mm^3^/min/W. The ablation threshold of dentin was significantly lower than that of enamel for both laser wavelengths. IR irradiation showed a nearly two times higher ablation efficiency in dentin than enamel, with 1.74 ± 0.05 mm^3^/min/W, while green irradiation showed a substantial increase of approximately 4.5 times, achieving 4.00 ± 0.05 mm^3^/min/W. No damage was observed by optical and scanning electron microscopic examination or by Raman spectroscopy for IR. However, modifications in the dentin structure occurred for fluence above 10 J/cm^2^ for green irradiation, which is well above the range of the optimal ablation of dentin.

To ensure optimal scanning efficiency, we explored the impact of pulse overlap on temperature measurements inside the tooth. Regimens with pulse overlaps were acceptable for both IR and green at 3.0 J/cm^2^ without inducing any structural changes or exceeding the recommended temperature limit of 5.5 °C^24^. However, using a higher laser fluence or pulse overlaps exceeding 75% may lead to charring, which should be avoided. Sufficient airflow directed to the ablation area can mitigate temperature-related concerns, as it consistently kept the temperature increment below 0.5 °C for both IR and green laser irradiation protocols.

In conclusion, this study demonstrates the importance of carefully selecting laser wavelengths for tooth ablation procedures. While UV demonstrated the lowest ablation threshold, this should be avoided as it causes substantial damage to tooth structures. IR and green wavelengths showed, overall, better performance, with green being particularly efficient for dentin ablation. The highest ablation rates can be achieved using green irradiation, but this would require dynamic control over the fluence when crossing the enamel and dentine boundary.

Moreover, using pulse overlapping and temperature control appropriately can further optimize the ablation process, ensuring safe and effective dental procedures. IR irradiation provided similar ablation rates for enamel and dentin in the range of laser fluence between 7 and 14 J/cm^2^, which would result in a constant ablation rate independent of the materials being ablated. It will thereby simplify control strategies, at the expense of maximal ablation rates.

In summary, we demonstrated that precise grooves can be performed in enamel and dentin with minimal temperature rise using IR and Green femtosecond laser wavelengths. Femtosecond lasers, being able to remove dental materials with sub-micron precision with the absence of damage to the underlying structures and without the need for water irrigation during the process^3^, offer several advantages over traditional dental instruments, promising benefits for both patients and dentists, resulting in improved health outcomes. Driven by wider adoption and research, we can expect femtosecond lasers will emerge as a viable alternative to mechanical drills in the future of dentistry^25^.

## Data Availability

The data that support the findings of this study are available from the corresponding authors upon reasonable request.
